# Mutational Meltdown in Primary Endosymbionts: Selection Limits Muller's Ratchet

**DOI:** 10.1371/journal.pone.0004969

**Published:** 2009-03-23

**Authors:** Julie M. Allen, Jessica E. Light, M. Alejandra Perotti, Henk R. Braig, David L. Reed

**Affiliations:** 1 Zoology Department and Florida Museum of Natural History, University of Florida, Gainesville, Florida, United States of America; 2 Florida Museum of Natural History, University of Florida, Gainesville, Florida, United States of America; 3 School of Biological Sciences, Plant Science Laboratories, University of Reading, Reading, United Kingdom; 4 School of Biological Sciences, University of Wales, Bangor, Gwynedd, United Kingdom; University of Exeter, United Kingdom

## Abstract

**Background:**

Primary bacterial endosymbionts of insects (p-endosymbionts) are thought to be undergoing the process of Muller's ratchet where they accrue slightly deleterious mutations due to genetic drift in small populations with negligible recombination rates. If this process were to go unchecked over time, theory predicts mutational meltdown and eventual extinction. Although genome degradation is common among p-endosymbionts, we do not observe widespread p-endosymbiont extinction, suggesting that Muller's ratchet may be slowed or even stopped over time. For example, selection may act to slow the effects of Muller's ratchet by removing slightly deleterious mutations before they go to fixation thereby causing a decrease in nucleotide substitutions rates in older p-endosymbiont lineages.

**Methodology/Principal Findings:**

To determine whether selection is slowing the effects of Muller's ratchet, we determined the age of the *Candidatus* Riesia/sucking louse assemblage and analyzed the nucleotide substitution rates of several p-endosymbiont lineages that differ in the length of time that they have been associated with their insect hosts. We find that *Riesia* is the youngest p-endosymbiont known to date, and has been associated with its louse hosts for only 13–25 My. Further, it is the fastest evolving p-endosymbiont with substitution rates of 19–34% per 50 My. When comparing *Riesia* to other insect p-endosymbionts, we find that nucleotide substitution rates decrease dramatically as the age of endosymbiosis increases.

**Conclusions/Significance:**

A decrease in nucleotide substitution rates over time suggests that selection may be limiting the effects of Muller's ratchet by removing individuals with the highest mutational loads and decreasing the rate at which new mutations become fixed. This countering effect of selection could slow the overall rate of endosymbiont extinction.

## Introduction

Primary endosymbiotic bacteria (p-endosymbionts) are thought to have enabled insects to become ecologically diverse by facilitating radiations into niches with nutrient–poor diets such as plant sap, wood, and vertebrate blood. P-endosymbionts live within specialized host organs called mycetomes and are transmitted transovarially (vertically) from mother to offspring [1]. Some p-endosymbionts are required for host reproduction [2], [3] whereas others provide essential services for their hosts such as light emission, or synthesis of amino acids, cofactors, and vitamins that are lacking in the host's specialized diet [4].

Because of their endosymbiotic lifestyle and strict vertical transmission, all p-endosymbionts share many characteristics such as small populations, reduced genomes, and AT bias [5]–[8]. P-endosymbionts also accrue slightly deleterious mutations at a faster rate than free-living bacteria [6]. This is thought to be due to genetic drift acting on already small populations that go through population bottlenecks at each host generation [9]. Furthermore, because p-endosymbionts are maternally transmitted, it is thought that recombination cannot occur between different strains [10]. The steady accumulation of these deleterious mutations is a process called Muller's ratchet [6], [11], [12].

Muller's ratchet states that in small populations, due to genetic drift, there is a chance that individuals with the fewest mutations will fail to reproduce [11], [13]. When this happens, the ratchet clicks [12] irreversibly increasing the overall mutational load of the population. As mutational load increases, the relative fitness decreases through reduced reproductive rate or reduced survivorship [14], [15]. If deleterious mutations continually get fixed over time, the p-endosymbiont may experience a mutational meltdown ultimately resulting in extinction [13].

It is thought that once a p-endosymbiont has deteriorated to the point of being nonfunctional, it may be replaced by another bacterium [16]. Evidence for p-endosymbiont replacement, however, is scarce, having only been found in a few species of aphids [17], [18], weevils [19], and more recently in sucking lice [20], [21]. In contrast, some insect/p-endosymbiont assemblages have existed for hundreds of millions of years without evidence of p-endosymbiont replacement, suggesting that Muller's ratchet may slow or stop over time.

Several mechanisms have been proposed to explain how Muller's ratchet might slow or stop over time. These mechanisms include back mutations [22], compensatory responses [23], and selection. The probability of a back mutation “correcting” each slightly deleterious mutation is so minimal compared to the probability of a forward mutation that it has been ignored in models of Muller's ratchet [22]. Compensatory responses have been suggested in the case of the GroEL protein. The GroEL protein mediates the folding of polypeptides, and it is found to be highly expressed in *Buchnera* and other p-endosymbionts. Up-regulation of the GroEL protein may reduce the effects of other slightly deleterious mutations that may change the folding of important proteins [6]. However, little is known about the overall effect of compensatory responses on Muller's ratchet. Selection may by acting to slow or stop Muller's ratchet through long term bottlenecks (which cause the variance in fitness to be increased among hosts for selection to act upon [24]), and through epistatic interactions between slightly deleterious mutations [25], [26] where the effect on fitness of the p-endosymbiont does not increase linearly with each mutation that becomes fixed in the population. Epistatic interactions may make the ratchet slow down but not necessarily stop [27]. In this study, we are specifically interested in determining if selection is acting to reduce the number of slightly deleterious mutations that become fixed in the population, thus slowing the process of Muller's ratchet in some insect/p-endosymbiont assemblages.

Early studies of non-synonymous nucleotide substitution rates suggested that selection was weak in p-endosymbionts [6], [28]. Recent studies of the genomes of the p-endosymbiont of aphids (*Buchnera*), however, show that selection may play a role in slowing genome degradation (i.e., gene loss) and AT bias. For example, Tamas et al. [29] found long-term genomic stasis in two *Buchnera* genomes that diverged around 50–70 Mya. They concluded that gene loss must have occurred early in the association between *Buchnera* and its host, only to stabilize later due to selective constraints. Early and rapid gene loss was also found in another lineage of *Buchnera* with divergence dates of 80–150 My [30], [31]. Clark et al. [7] suggested that selection might also reduce AT bias over time in *Buchnera*, and therefore slow the speed of Muller's ratchet.

Selection could decrease the rate at which slightly deleterious mutations become fixed in the population, especially as the p-endosymbiont/insect association ages. As the mutational load increases in p-endosymbionts, selection may act to remove individuals with the highest mutational loads (i.e., the least functional individuals). This would then slow the rate of fixation of slightly deleterious mutations, which would result in a reduction in the overall nucleotide substitution rate.

Early estimates of nucleotide substitution rate in p-endosymbionts consistently averaged 1–2% per 50 My [32]. However, recent studies have documented much faster evolving p-endosymbionts [33], [34]. The fastest rate reported to date is 33.5% per 50 My for *Candidatus* Riesia (hereafter *Riesia*), the p-endosymbiont of primate sucking lice (Anoplura: Pediculidae and Pthiridae) [34]. The p-endosymbionts with the highest nucleotide substitution rates appear to be among the youngest insect/p-endosymbiont associations, which suggest that rates may vary in relation to the age of the association. However, the age of the association of *Riesia* with its host is unknown. Therefore, we first determine the age of the association of *Riesia* with its host and calculate more rigorously the rate of molecular evolution for *Riesia*.

Additionally, we estimate the nucleotide substitution rates from the *16S* ribosomal DNA gene (*16S rDNA*) for a diverse assemblage of p-endosymbionts to test the prediction that selection reduces the effect of Muller's ratchet over time. If selection slows Muller's ratchet over time, then we should observe an inverse relationship between nucleotide substitution rates and the age of the insect/p-endosymbiont assemblage. A decline in substitution rates would be consistent with an increase in selection over time. The mutational meltdown model predicts that given no opposing force, p-endosymbionts should steadily accrue slightly deleterious mutations until extinction. An increase in selection over time might allow p-endosymbionts to stave off extinction, which would explain the existence of ancient insect/p-endosymbiont associations.

## Results

### Age of *Riesia*/Louse Association

**Figure 1:**
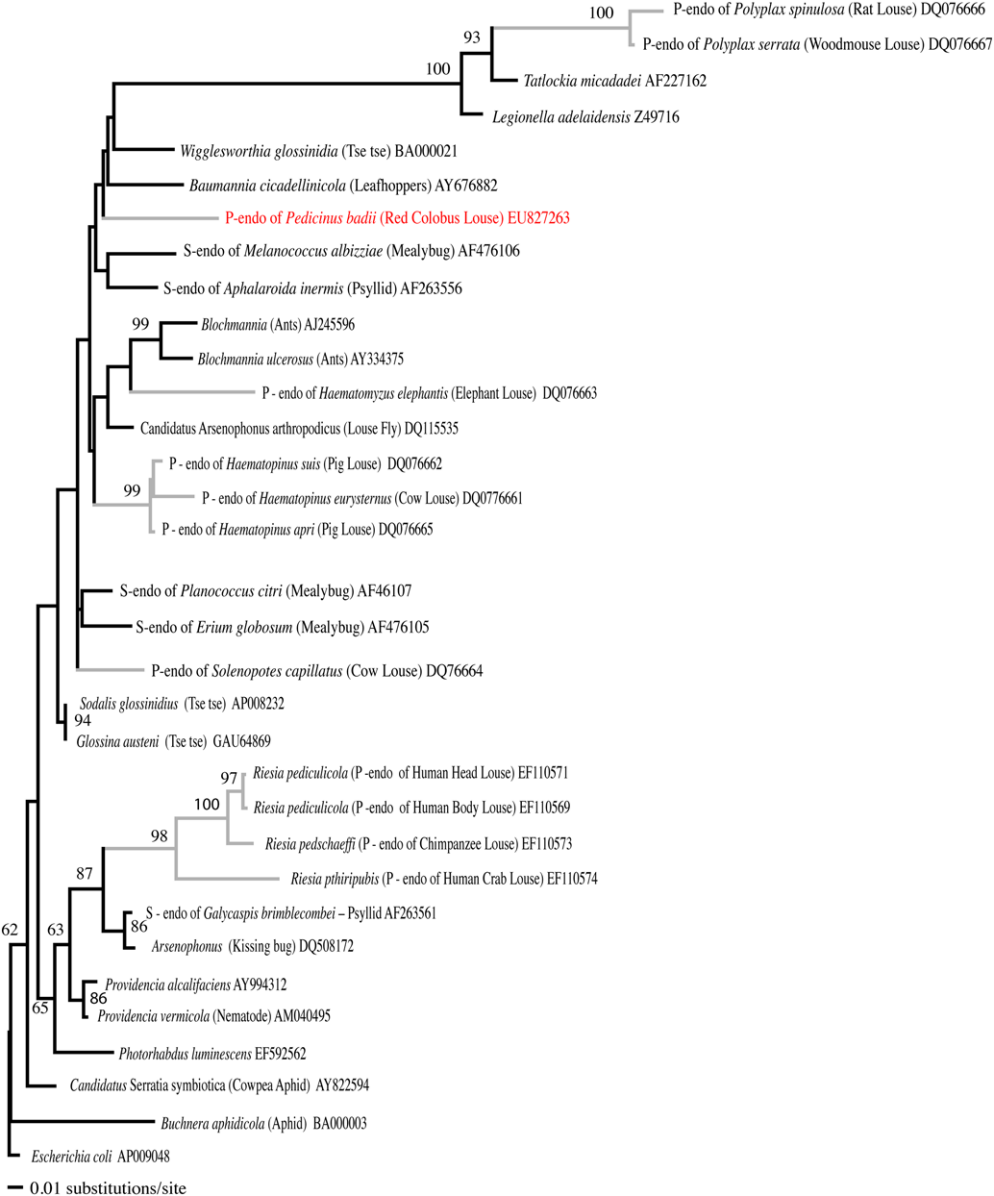
Maximum likelihood phylogram representing phylogenetic relationships of louse p-endosymbionts, common insect p-endosymbionts, and closely related taxa as determined from a BLAST search of each louse endosymbiont sequence. Numbers at nodes indicate maximum likelihood support values greater than 60. Gray lines indicate louse p-endosymbionts. The p-endosymbiont from *Pedicinus badii* (the louse that parasitizes Red Colobus monkeys) is shown in red demonstrating that it does not group with the *Riesia* p-endosymbionts. There are now at least six distinct clades of p-endosymbionts sampled from sucking lice.

The age of the association between the fast-evolving p-endosymbiont *Riesia* and the primate sucking lice in which it lives was previously unknown. In order to estimate this age, we examined the p-endosymbiont from a closely related louse genus, *Pedicinus*. The p-endosymbiont from *Pedicinus badii* (a louse that parasitizes Old World monkeys) does not group with the anthropoid primate louse p-endosymbionts (the *Riesia* lineage) in our Maximum Likelihood or Bayesian (not shown) phylogenetic analyses (Figure 1). The Maximum Likelihood analysis groups the p-endosymbiont of *Pedicinus badii* at the base of a clade containing the p-endosymbionts *Wigglesworthia* and *Baumannia* (p-endosymbionts of tse-tse flies and leafhoppers, respectfully), some free-living bacteria, and the p-endosymbionts of distantly related sucking lice of rodents (Figure 1). Bayesian phylogenetic trees were largely identical, and placed the p-endosymbiont of *Pedicinus badii* at the base of the same clade. Analyses constraining the *Pedicinus* p-endosymbiont to group with the *Riesia* lineage produced trees that were significantly worse than the best Maximum Likelihood tree according to the Kishino-Hasegawa (*p* = 0.004) and Shimodaira-Hasegawa (*p* = 0.004) tests. Furthermore, none of the suboptimal trees from the Bayesian analysis were consistent with this topological constraint (*p*<0.001). We can therefore formally reject the hypothesis that the p-endosymbiont sequences from *Pedicinus badii* are sister to or embedded within the *Riesia* lineage. Because *Pedicinus* is the closest living relative of *Pediculus* and *Pthirus*, this phylogenetic analysis demonstrates that the age of the association between *Riesia* and primate lice has an upper bound at 25 My for the split between *Pedicinus* and *Pediculus* and *Pthirus*. Thus, the age of association between *Riesia* and their louse hosts is between 12.95 and 25 My, making this one of the youngest insect/p-endosymbiont assemblages known.

### Absolute Rates

**Figure 2:**
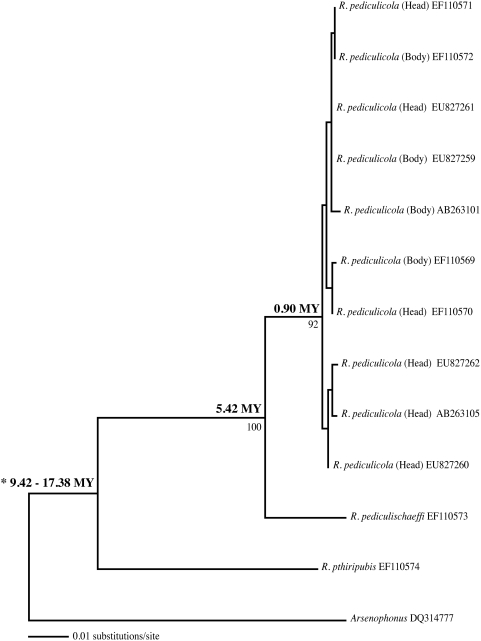
Maximum likelihood phylogram representing phylogenetic relationships of *Riesia* p-endosymbionts. Numbers above the node represent divergence dates (in millions of years) whereas numbers below the nodes are bootstrap support values (only numbers greater then 60 are shown). The divergence date calibration point of 9.42–17.38 My is indicated with an asterisk.

Using the 9.42–17.38 My split between *Pediculus* and *Pthirus* as a calibration date [35], [36], we estimated the divergence time between *Riesia pediculicola* (human head and body louse p-endosymbionts) and *Riesia pediculischaeffi* (chimp louse p-endosymbionts) at 5.42 My, which is very close to the ages estimated for these lice and for their vertebrate hosts [36]. We further estimate that the p-endosymbionts of the human head lice originated 0.90 My (Figure 2), which is similar to the estimate of 1.2 My for the lice [36]. The pairwise sequence divergence for *Riesia* p-endosymbionts of *Pediculus* and *Pthirus* is 12.90% (GTR+I model), therefore the absolute rate of evolution of *Riesia* p-endosymbionts is 0.0037–0.0684 substitutions per site per million years which translates to 18.56–34.24% per 50 My (Table 1).

**Table 1:** Percent sequence divergence and rate of nucleotide substitution of the *16S rDNA* of *Riesia* the p-endosymbiont from anthropoid primate lice calibrated at 9.42 and 17.38 My.

	9.42 My	17.38 My
**Substitutions / My**	0.0137	0.0074
**Substitutions / site / My**	0.0068	0.0037
**Percent / 50 My**	34.24%	18.56%

### Substitution rates among host/endosymbiont lineages

**Figure 3:**
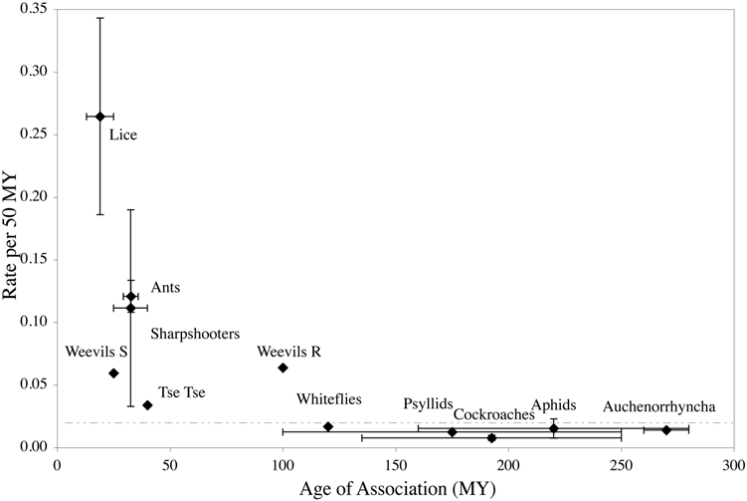
Primary endosymbiont nucleotide substitution rates as a function of the age of insect/p-endosymbiont association. Ages and rates were retrieved from the literature or were calculated from pairwise sequence divergences and are shown as percent per 50 My. Point estimates (diamonds) represent the median value between the upper and lower estimates (bars show the full range of dates). The dotted line indicates a rate of 2% per 50 My. It has been suggested that the tse-tse fly and aphid p-endosymbionts (*Wigglesworthia* and *Buchnera*, respectively) are closely related to each other and are the result of a more ancient endosymbiosis event than represented here [68], [69]. Using an older date to calculate rates, however, does not change the results (data available upon request).

**Figure 4:**
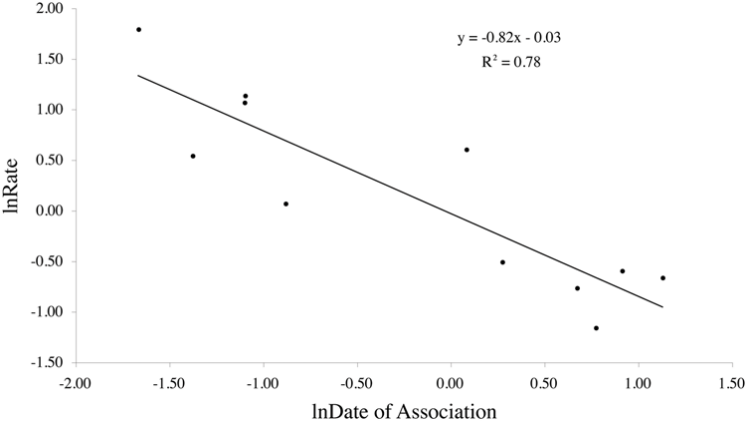
Reduced major axis regression of the log transformed data from Figure 3. The age of the association is on the x-axis and the rate of nucleotide evolution is on the y-axis. 78% of the variation in rate of nucleotide evolution can be explained by the age of the association suggesting that the rate of nucleotide evolution does decrease over time in p-endosymbionts of insects.

When the rates of nucleotide substitution for *Riesia* are compared to other known insect/p-endosymbiont systems, we find that the rate of substitutions in *16S rDNA* decreases with age of association and levels off after 100 My (Figure 3). Although the majority of the systems are evolving at a rate similar to what was reported for *Buchnera* (1–2% per 50 MY), the younger systems are evolving much faster (3–34% per 50 MY; Figure 3). Reduced major axis regression of the log-transformed data indicates that 78% of the variation in rates of nucleotide evolution can be explained by the age of the association (Figure 4) and that the decrease in rates is exponential. The pairwise sequence divergence in *Riesia* calculated here (18.56–34.24% per 50 My) were corrected with a best-fit model of nucleotide substitution. Some previous studies did not use the best-fit evolutionary model to correct for multiple substitutions. Therefore, to test the impact of the substitution model, we also evaluated the same pairwise divergences using the Jukes-Cantor model. The Jukes-Cantor distances still provide a much faster rate of nucleotide substitution in *Riesia* (12.9–23.9% per 50 My), and it is important to note that this more simplistic model of molecular evolution underestimates the substitution rate by 30%.

## Discussion

In this study, we find that there is considerable variation (15 to 30-fold) in the rate of p-endosymbiont nucleotide evolution for the *16S rDNA* gene. The association between anthropoid primate lice and their p-endosymbionts in the genus *Riesia* represents one of the youngest insect/p-endosymbiont assemblages known to date (between 12.95 and 25 Ma), and compared to other insect/p-endosymbiont assemblages, *Riesia* is experiencing the highest rate of nucleotide substitution yet measured (18.56–34.24% per 50 My; Figures 3 and 4). Among all insect/p-endosymbiont assemblages examined, we find that 78% of the variation in nucleotide substitution rate can be explained by the age of the association (Figure 4). Higher rates of nucleotide substitution are associated with the youngest host/p-endosymbiont assemblages despite correcting for multiple substitutions (Figures 3 and 4). Nucleotide substitution rates decrease to approximately 1–2% per 50 My when the insect/p-endosymbiont assemblages reach approximately 100 My of age. These findings are consistent with the hypothesis that selection reduces the effect of Muller's ratchet over time.

An alternative explanation is that substitution rate variation is driven by variation in p-endosymbiont population size (i.e., smaller populations evolve faster than larger ones due to genetic drift). Estimates of effective population size are not available for the taxa used in this study, therefore this cannot be tested directly. It is likely that p-endosymbiont effective population size is governed largely by host effective population size, transmission dynamics, and other aspects of the p-endosymbiont/host relationship. Additional research, however is needed to directly test these hypotheses.

As proposed in the mutational meltdown model, selection is likely the force reducing the number of deleterious mutations that become fixed in p-endosymbiont populations. The slow but steady accumulation of deleterious mutations is predicted to impair the p-endosymbiont's ability to function if the process of Muller's ratchet goes unchecked. Our data suggest that selection may steadily grow stronger in older assemblages and thereby slow the rate of Muller's ratchet by removing individuals with the highest mutational load. An increase in selection over time explains why some p-endosymbionts have ancient associations with their insect hosts and remain functional for hundreds of millions of years. However, younger assemblages do not always have a higher rate, especially among co-endosymbionts. Weevils (Insecta: Coleoptera) have two lineages of p-endosymbionts (termed the R- and S-clades) that are evolving at roughly the same rate even though R-endosymbionts have been associated with their hosts for 75 My longer [19]. The rate differences between weevil p-endosymbionts, however, are minimal and fit well within the limits of other p-endosymbionts (Figure 4).

It has been predicted that selection plays a major role in slowing down or stopping Muller's ratchet. As slightly deleterious mutations go to fixation, they reduce the fitness of the host. If there were synergistic epistatic interactions between mutations we would expect an exponential increase in selection over time, which is consistent with our data.

### Endosymbiosis

The importance of p-endosymbionts to insects, concerning their radiation into nutrient poor niches, cannot be overstated [1], [10], [21]. Yet, very little is known about how bacteria become endosymbionts, although it is thought that they might originate from attenuated pathogens [37]–[40]. Regardless of the mechanism, the basic requirements for becoming an endosymbiont are substantial. The endosymbiont must overcome many host physical, cellular, and molecular barriers for internalization [41], and a mechanism must develop for transmission of the bacteria to the insect's offspring [5]. Within the *Riesia* lineage alone, these bacteria undergo two extra-cellular migrations and are housed in no fewer than four distinct mycetomes [42]. From an evolutionary perspective this complex host/p-endosymbiont interaction seems highly specialized and the likelihood of repeated endosymbiont replacement over time is unknown.

If slightly deleterious mutations were to continue unabated in insect/p-endosymbiont associations, then we would expect to see a steady increase in the number of nucleotide substitutions over time, maintaining a high rate of molecular evolution. Instead we see a decline in the substitution rate (Figure 3). Our interpretation is that as the host/p-endosymbiont association ages, and the mutational load of p-endosymbionts increases, the role of selection increases and slows the rate of accumulation of slightly deleterious mutations. This is consistent with the studies of Tamas et al. [29], van Ham et al. [30] and Clark et al. [7] who found that the rate of genome degradation and AT bias also decreases over time. Our findings are also consistent with that of Delmotte et al. [43], who found that the genes lost at the beginning of the association were those that were the least selectively constrained. We propose that there are selective constraints embodied in the process and maintenance of endosymbiosis that could mitigate the effects of Muller's ratchet in late-stage or well-established endosymbionts. Bergstrom and Pritchart [24] suggested that long-term bottlenecks increase the selection pressure on deleterious mutations by increasing the variance in fitness among hosts. Therefore host-level selection may help to maintain the endosymbiosis over the long-term.

Although our data show that Muller's ratchet slows through time, Muller's ratchet may not cease to act entirely in p-endosymbionts, and three outcomes have been recorded. An endosymbiont may become so degraded that it effectively becomes an organelle such as *Carsonella*, the p-endosymbiont of psyllids [44]. *Carsonella* has been associated with its host for 100 to 250 My [45] and has a low rate of nucleotide evolution (Figure 3). It may be possible that *Carsonella* remains functional only because many of its genes have been transferred to the host genome and the products of these genes are shipped back to the symbiont [10]. Alternatively, the biological functions of an endosymbiont may be so reduced that a second endosymbiont is required. This is the case with the co-primary endosymbionts *Baumannia* and *Sulcia* [46], which have lost so many metabolic genes that by themselves they would not be viable or functional as endosymbionts [46]. *Sulcia*, the more ancient p-endosymbiont, has been associated with its host for 250 My and has a genome size of 245 kb [46] whereas *Baumannia*, the younger p-endosymbiont, has only been associated with sharpshooters for 25–40 My [47] and has a larger genome of ∼686 kb [48]. These co-primary endosymbionts only survive by complementing each other. Finally, an endosymbiont may become so degraded it is eventually replaced, possibly out-competed, by another bacterial lineage. In fact, Anderson and Kurland [16] suggested that obligate bacteria may replace each other at rates determined by Muller's ratchet. The gradual accumulation of slightly deleterious mutations, slowly degrading the genome over time, may make the endosymbiont unable to compete with relatively benign pathogens that have the ability to participate in the mutualism. These less-attenuated pathogens could then replace the older degraded p-endosymbiont lineages, which may have been the case with some aphid lineages [17], [18], weevils [19], and sucking lice [20]. The relationship of the pathogen and host at this point would change to a mutualistic one thereby giving the new bacterial lineage the benefits of this relationship such as potentially escaping host immune defense through provision of various host transported mycetomes that protect the new p-endosymbiont, which has been found in lice for many stages [42]. For the new mutualist, however, in some cases this new arrangement might hasten its extinction as Muller's ratchet engages.

## Materials and Methods

### 1. Age of the Riesia/Louse Assemblage:

The oldest split found within *Riesia* dates to 12.95 Ma and occurs between p-endosymbionts associated with the louse genera *Pediculus* and *Pthirus* [34]. However, some of the oldest divergences among related lice (those of the genus *Pedicinus*; Anoplura: Pedicinidae) date back to the split between their Anthropoid and Cercopithecoid primate hosts, ca. 25–30 Ma. At present it is not known whether lice of the genus *Pedicinus* carry the *Riesia* lineage of p-endosymbionts. Therefore, we have molecularly characterized the endosymbiont of *Pedicinus* to evaluate the age of the *Riesia*/louse association. If the p-endosymbiont in the louse genus *Pedicinus* does not belong to the *Riesia* lineage, then the *Riesia*/louse association is between 12.95 and 25 My.

#### Specimen Collection and DNA Sequencing

To determine the age of the *Riesia*/louse association, specimens of *Pedicinus badii* were collected from Red Colobus monkeys (*Procolobus rufomitratus*) from Kibale National Park in Uganda. Three human head louse specimens (*Pediculus humanus capitis*) and a single body louse specimen (*Pediculus humanus humanus*) were collected from individuals in West Palm Beach, Florida, USA, and the rabbit-adapted strain held at the Insect Control and Research Lab in Maryland, USA, respectively, to determine the absolute rate of nucleotide substitution in *Riesia*. Whole lice were washed twice with 400 µl saline EDTA, 15 µl of 20% SDS and 5 µl lysozyme to remove any external bacteria. The sample then was crushed and genomic DNA was isolated using the DNeasy Tissue Kit (QIAGEN Inc., Valencia, California). PCR amplification of the endosymbiont *16S rDNA* gene (1.5-kbp) was performed in 25 µl reactions with primers 27F (5′ – AGA GTT TGA TCC TGG CTC AG – 3′) and 1392R (5′ – CAC GGA TCC ACG GGC GGT GTG TRC – 3′) for the *Pedicinus* endosymbiont, and the *Riesia* specific primer 461F (5′ – ACA GAA GAA GCA CCG GCT AA – 3′) and general reverse primer 1525R (5′ – AGA AAG GAG GTG ATC CAG CC – 3′) for *Pediculus* endosymbionts. Each amplification was performed using standard reaction conditions with 10 ng of template DNA, 300 nM of each primer, 200 µM of each dNTP, 2.5 mM MgCl_2_ and 0.02 U of Taq DNA polymerase (Promega, Madison, Wisc.) per µl of reaction mix. Cycling conditions consisted of an initial denaturation step (94°C, 10 min), 30 cycles of amplification involving denaturation (94°C, 1 min), annealing (50–52°C, 1 min) and extension (65°C, 1 min), and a final extension step at 65°C for 10 min. The *16S rDNA* PCR product was purified with ExoSAP-IT (USB Corporation) and then cloned into the pTOPO 4.0 vector (Invitrogen). Recombinant clones were sequenced in both directions at the University of Florida sequencing facility using vector-specific primers and internal sequencing primers as in Reed and Hafner [49]. Sequences were edited using Sequencher Version 4.1 (Gene Codes Corporation, Ann Arbor, Michigan) and deposited in GenBank (Accession numbers: EU827259–EU827263 *Reisia pediculicola* from *Pediculus humanus humanus*, three sequences of *Riesia pediculicola* from *Pediculus humanus capitis* and the primary-endosymbiont from *Pedicinus badii*, respectively).

#### Phylogenetic analysis

Phylogenetic analyses were used to determine the placement of the *Pedicinus* p-endosymbiont with respect to other known bacteria, and to estimate the age of the *Riesia*/louse association. The *16S rDNA* sequence of the *Pedicinus* p-endosymbiont obtained above was compared to 32 bacterial *16S rDNA* sequences downloaded from GenBank which included louse p-endosymbionts, insect p-endosymbionts, free-living *Escherichia coli*, and sequences with the highest sequence similarity to the *Pedicinus* and other louse p-endosymbionts obtained form GenBank BLAST searches (Table 2). All sequences were aligned using Clustal X [50], then manually adjusted by eye.

**Table 2:** Bacteria taxa, their hosts, and GenBank accession numbers used in the phylogenetic analysis presented in Figure 1.

Endosymbiont	Host Species	GenBank
*Riesia pediculicola*	*Pediculus humanus capitis*	EF110571
*Riesia pediculicola*	*Pediculus humanus humanus*	EF110569
*Riesia pediculischaeffi*	*Pediculus schaeffi*	EF110573
*Riesia pthiripubis*	*Pthirus pubis*	EF110574
—	*Haematomyzus elephantis*	DQ076663
—	*Haematopinus apri*	DQ076665
—	*Haematopinus eurysternus*	DQ076661
—	*Haematopinus suis*	DQ076662
—	*Solenopotes capillatus*	DQ076664
—	*Polyplax spinulosa*	DQ076666
—	*Polyplax serrata*	DQ076667
*Blochmannia ulcerosus*	*Camponotus* ants	AY334375
*Blochmannia sp.*	*Camponotus* ants	AY334375
*Sodalis glossinidius*	*Glossina*	AJ245596
*Baumannia cicadellinicola*	leafhoppers	AY676882
*Arsenophonus sp.*	*Triatoma melanosoma*	DQ508172
*Buchnera aphidicola*	*Acyrthosiphon pisum*	BA000003
*Wigglesworthia glossinidia*	*Glossina brevipalpis*	BA000021
—*	*Melanococcus albizziae*	AF476106
—*	*Planococcus citri*	AF476107
—*	*Erium globosum*	AF476105
—*	*Aphalaroida inermis*	AF263556
—*	*Glycaspis brimblecombei*	AF263561
—*	*Glossina austeni*	GAU64869
—*	*Planococcus citri*	AF476107
Serratia symbiotica*	*Aphis craccivora*	AY822594
*Tatlockia micdadei*	–NA–	AF227162
*Legionella adelaidensis*	–NA–	Z49716
*Photorhabdus luminescens*	–NA–	EF592562
*Providencia alcalifaciens*	–NA–	AY994312
*Providencia vermicola*	*Steinernema thermophilum*	AM040495
*Arsenophonus arthropodicus*	*Pseudolynchia canariensis*	DQ115535
*Escherichia coli*	–NA–	AP009048

Some bacteria lack a species name because they have not been characterized completely. Secondary rather than primary endosymbionts are indicated with an asterisk. Free-living bacteria have no associated host.

Modeltest v. 3.7 [51] was used to determine a model of nucleotide evolution according to an Akaike Information Criterion (GTR+I+G; [52], [53]). This best-fit model was used in Maximum Likelihood (ML) and Bayesian phylogenetic analyses performed in PAUP*4.0b10 and MrBayes 3.12 [54], [55], respectively.

For the ML analyses, full heuristic ML and bootstrap (200 pseudoreplicates) searches were conducted with 10 random addition replicates and tree bisection-reconnection branch swapping using the best-fit model in PAUP* 4.0b10 [55]. In the Bayesian analyses, model parameters were treated as unknown variables with uniform priors and were estimated as part of the analysis. Bayesian analyses were initiated with random starting trees, run with four incrementally heated chains (Metropolis-coupled Markov chain Monte Carlo; [54] for 10 million generations, and sampled at intervals of 1000 generations. Two independent Bayesian analyses were run to avoid entrapment on local optima. Stationarity was assessed by plotting the log-likelihood scores of sample points against generation, and a conservative burn-in period of 25% was discarded. The retained equilibrium samples were used to generate a 50% majority rule consensus tree with the percentage of samples recovering any particular clade representing that clade's posterior probability [54].

Alternative phylogenetic hypotheses were compared statistically using the Kishino-Hasegawa (KH) and the Shimodaira-Hasegawa (SH) tests as implemented in PAUP*4.0b10 (MP and ML analyses using RELL optimization and 1,000 bootstrap replicates; [56]–[58]). Suboptimal trees from the Bayesian analyses also were examined to assess alternative phylogenetic hypotheses. The frequency of the Markov chain Monte Carlo trees in agreement with an alternative hypothesis equals the probability of that alternative hypothesis being correct [59]. The probability of trees agreeing with alternative subfamily hypotheses was calculated by applying constraint-based filter trees implemented in PAUP*4.0b10 [55], [59].

#### Absolute Rates of Nucleotide Evolution in *Riesia*

To determine the absolute rate of nucleotide substitution in *Riesia*, the *16S rDNA Riesia* sequences obtained above were aligned with p-endosymbiont sequences of human head lice (*R. pediculicola*; GenBank Accession Numbers AB263105, EF110570, and EF110571), human body lice (*R. pediculicola*; EF110569, EF110572, and AB236101), chimpanzee lice (*R. pediculischaeffi*; EF110573), and human pubic lice (*R. pthiripubis*; EF110574). Sequences were aligned using Clustal X [50] and manually adjusted using MacClade v. 4.06 [60]. These closely related sequences were easily aligned by eye with no ambiguity as to positional homology. Modeltest v. 3.7 [51] was used to determine a model of nucleotide evolution (GTR+G) for the *Riesia 16S rDNA* data as described above. A branch and bound ML analysis with a subsequent bootstrap analysis (200 replicates) was conducted using the best-fit model in PAUP* 4.0b10 [55].

Reed et al. [35] estimated divergence dates in the phylogenetic tree of primate lice, and estimated the split between the genera *Pediculus* and *Pthirus* to be 9.42–17.38 Ma ago. Because this node in the louse tree has a corresponding node of cospeciation in the endosymbiont tree [34], we are able to calculate an absolute rate of nucleotide substitution within *Riesia*, using the calibration range of 9.42–17.38 Ma for the split between *Pediculus* and *Pthirus* endosymbionts. Divergence times were estimated using penalized likelihood (TN algorithm) in the program r8s [61]. A smoothing parameter of 0.32 was determined using the cross-validation procedure.

### 2. Substitution rates among host/p-endosymbiont lineages

To determine whether the age of the host/endosymbiont association correlates with nucleotide substitution rate, we retrieved data from the literature of insect/p-endosymbiont assemblages having both estimates of the age of the association (through fossil evidence) as well as either rates of p-endosymbiont nucleotide evolution for *16S rDNA* or pairwise sequence divergences. In the absence of pairwise sequence divergences for a particular assemblage, we estimated these values by examining the two most divergent sequences as in Ochman et al. [32]. The systems examined included primate lice and *Riesia* [34], aphids and *Buchnera* [62], cockroach/termites and *Blattabacterium* [63], whiteflies and *Portiera* [64] (date from Poinar [65]), tse-tse flies and *Wigglesworthia* [66], Auchenorrhyncha (cicadas, hoppers and spittlebugs) and *Sulcia* [67], psyllids and *Carsonella* [45], weevils and *Nardonella* [19], weevils and the S-clade of p-endosymbionts [19], and ants and *Blochmannia* [33].

Rates of nucleotide substitution were plotted against the age of host/endosymbiont association. Because there is error in both the estimate of the rate of nucleotide evolution and the age of association, a reduced major axis regression was performed on the log-transformed data to better estimate the relationship between the age of the association and rate of nucleotide evolution in these systems.
